# Are medical students happy despite unhappy conditions: a qualitative exploration of medical student cohorts during disruptive conditions

**DOI:** 10.1186/s12909-023-04203-6

**Published:** 2023-04-05

**Authors:** Stephen Esguerra, Fiona Thuy Chiu, Alyssa Espinoza, Dan Williams, Amy Clithero-Eridon

**Affiliations:** 1 University of New Mexico, Albuquerque, USA; 2 Family & Community Medicine, University of New Mexico, PhD University of New Mexico School of Medicine, 1 UNM, Albuquerque, NM MSC09-5040, 87131 USA

**Keywords:** Medical student education, Burnout, Professional identity, Happiness, COVID-19

## Abstract

**Background:**

Shortly after the World Health Organization declared the SARS-CoV-2 outbreak a worldwide pandemic, medical school governing bodies issued guidance recommending pausing clinical rotations. Prior to the availability of COVD-19 vaccines, many schools implemented exclusively online curriculums in the didactic and clinical years. These unprecedented events and paradigm changes in medical education could contribute to trainee burnout, wellness, and mental health.

**Methods:**

This single-institution study interviewed first, second, and third-year medical students from a medical school in the southwestern United States. A semi-structured interview was conducted with paper-based Likert scale questions rating perceived happiness were administered both at the time of the interview and one year later in order to understand how their student experience and happiness were impacted. In addition, we asked participants to describe any major life events they experienced since the first interview.

**Results:**

Twenty-seven volunteers participated in the original interview. Twenty-four from the original cohort participated in the one-year follow-up. Happiness as a sense of self and who you “should be” was challenged during the pandemic and changes in happiness over time were not systematic across classes. Stress was caused not only by the pandemic which was experienced by all, but by a tripartite state of individual circumstances, academic workload requirements, and the world at large. Primary themes from the interviews were clustered around the individual, learner, and future professional levels and focused on the primacy of relationships, emotional wellness, stress management, professional identity, and impacts of educational disruptions. These themes created risk factors for developing imposter syndrome. Students demonstrated resiliency across cohorts and were able to utilize a variety of strategies to achieve and maintain both physical and mental health, but the primacy of relationships both personally and professionally was noted.

**Conclusion:**

Medical students’ identities as individual persons, a learner, and future medical professionals were all impacted by the pandemic. The results from this study suggest that the COVID-19 pandemic and changes in the learning format and environment may create a new risk factor in the development of imposter syndrome. There is also an opportunity to re-consider resources to achieve and maintain wellness during a disrupted academic environment.

## Background

In 2020, amid rising COVID-19 cases and the declaration of a pandemic by the World Health Organization (WHO), numerous medical schools made the decision to convert in-person didactics and clinical rotations to virtual platforms and remote educational activities[1]. In the United States, this change was in part based on the guidance of the American Academy of Medical Colleges and Liaison Committee on Medical Education recommending the withdrawal of students from in-person activities at the onset of the pandemic [2, 3]. For the last decade, the preclinical years had already been heavily incorporating asynchronous learning into the curriculum, allowing students to learn from outside the classroom with the exception of some group activities [4, 5]. However, with the circumstances surrounding the largely unknown characteristics of SARS-Cov-2 transmission at the start of the pandemic, the year 2020 was the first time in which in-person learning was completely absent in many medical schools. This change was particularly extraordinary for medical students transitioning to their clinical years, whose roles in caring for patients were drastically altered in the online environment.

Prior to the COVID-19 pandemic, higher rates of burnout, stress, and stress-related mental health problems in medical students compared to their age-matched, non-medical peers had been well-documented in the literature [6–10]. The pre-COVID-19 prevalence of depression in medical students has been estimated to be greater than 27% with approximately 11% reporting suicidal ideation [11, 12]. The rates of burnout have been described to affect 40% to greater than 50% of the medical student population with risk factors including minority race, disability, debt burden, alcohol/substance use, and being out-of-phase with the medical school curriculum [12–16]. The pandemic-related transition to primarily online learning was a disruptive and sudden occurrence that may have exacerbated stress levels in this already vulnerable population. Emerging data on medical students’ responses regarding the impact of these teaching and learning modifications have been mixed with positive aspects including flexibility in coursework and negative aspects including increased mental health problems, feelings of isolation, increased sleep, poor dietary habits, reduced physical exercise, decreased social relationships, concerns about graduating on time and residency applications, and reduced knowledge and preparedness [17–27]. These data emphasize the need to examine the tripartite state of wellness, satisfaction with education, and subjective happiness of medical students for a comprehensive overview of general well-being during this challenging time.

At the study site, students undergo three phases during their medical school training (Phase I, II, and III). Phase I involves basic science didactics traditionally taught in a classroom-based format culminating in students taking the United States Medical Licensing Exam (Step 1) typically by the end of their second year. After passing Step 1, students transition to Phase II which involves clinical rotations in multiple specialties. These specialties are Family Medicine, Pediatrics, Internal Medicine, Surgery, Obstetrics/Gynecology, Neurology, and Psychiatry. At the end of each rotation, medical students take a standardized shelf exam that tests the clinical knowledge they should have obtained from that particular rotation.

Students then transition to Phase III during their fourth year. Phase II students interrupted by COVID were transitioned to a hybrid curriculum consisting of six months of seven three-week virtual clerkships followed by six months of clinical clerkships. Phase I students were completely taken out of the classroom and transitioned to a virtual curriculum. Similar innovative changes in the medical school learning environment, such as transitions to virtual formats, modified calendars, and new electives were also taking place at other institutions to address the challenges presented to medical educators [3, 28–32]. Unsurprisingly, the novelty and uncertainty arising from these abrupt changes led to uncertainty and frustration among many medical students.[33, 34] We aimed to evaluate how the subjective happiness of medical students changed over the course of a year in the pandemic.

## Methods

### Study population

To achieve these aims, we used a mixed methods cohort study that including qualitative interviews and a pre-post survey measure of wellbeing. We used a simple random sampling technique by sending an email to all first, second, and third year medical students at our institution. We selected the first 10 respondents for years one and two. There were seven respondents from year 3. Participants were interviewed using a standardized script by three student researchers (S.E., F.C., and A.E.) not in the same educational year as a participant. The students were given training on interview techniques by the senior researcher (A.C.E.) Fourth year medical students were excluded from the study due to scheduling conflicts and impending transition to residency. Participants were given a merchandise card in the amount of $25 as an incentive to participate.

### Data collection

We collected data using an interview script. Participants completed a one-to-one interview with one of the primary investigators through a HIPAA-compliant zoom meeting. Interviewers read each question to the interviewee and consent was obtained prior to each interview. Participants received a copy of the consent form which outlined the purpose of the study prior to their scheduled interview. Interviews lasted approximately 45 min to 1.5 h and were all conducted in English. Primary investigators recorded responses verbatim during the interviews on their laptops which were stored in a password secured cloud repository. Participants could decline to answer any questions.

We collected demographic information including age, gender, ethnicity, relationship status, number of children, parental care-giving responsibilities, and current living situation. We combined ethnicity in the total column for clarity, but accounted for variation of self-identification in the individual year columns.

We asked students to rate their subjective happiness using a validated scale that correlates with other existing happiness measures.[35] Using a Likert scale from 1 to 7 (1 – Completely dissatisfied, 2 – Mostly dissatisfied, 3 – Somewhat dissatisfied, 4 – neither satisfied nor dissatisfied, 5 – Somewhat satisfied, 6 – Mostly satisfied, and 7 – Completely satisfied). We also asked participants about their satisfaction with the number of hours spent on Zoom since the beginning of their online curriculum, satisfaction with the school’s approach to helping with wellness during the pandemic, satisfaction with synchronous learning, and satisfaction with asynchronous learning. Synchronous learning referred to lectures and group activities given within a designated time period. Asynchronous learning consisted of independent studying material such as pre-recorded lectures or assigned reading material. Curriculum satisfaction questions were not validated as the rapid transition to on-line learning and initiation of wellness activities during COVID was still a new phenomenon.

Additionally, we asked participants to describe how the pandemic affected them personally, professionally, and academically. We queried participants on whether their wellness changed since the start of the pandemic. We assessed the awareness and utilization of wellness programs available through the medical school along with eliciting suggestions for improvement in this area. We further asked participants about stressful events that occurred since the start of the pandemic and the positive and/or negative coping mechanisms they used for stress management.

### Post-survey

All participants were offered the opportunity to participate in a follow-up survey administered one year after the initial interview. Students received a link to the online survey and responses were associated with their same de-identified number from the initial survey. In the post-survey, we asked participants to rate their subjective happiness using the same validated scale in the first survey. Students were not provided their original responses. We also asked participants to describe any major life events they have experienced since the first interview. We defined the post-survey as being “post-Covid” as a vaccination was available and restrictions were being lifted.

### Data analysis

We used a qualitative thematic analytic approach. Participants received a copy of their responses at the end of the interview through email for accuracy verification. They were given five days to approve the transcript or make any changes. Questionnaire responses were linked by a number 1 to 27 in order to maintain participant anonymity. Themes were inductively coded by the researchers who agreed on themes via a consensus approach. Descriptive statistics are reported to give context to the findings. Tests of statistical significance were not performed due to lack of power.

### Ethics approval and consent to participate

The University of New Mexico Human Research and Review Committee approved this study (HRRC#21–045). This study was conducted in accordance with the guidelines in the Helsinki Declaration of 2013. Informed verbal consent was obtained prior to interviews taking place. Procedure for informed verbal consent was approved by the University of New Mexico Human Research and Review Committee.

## Results

### Student population and demographics

Twenty-seven students participated in the initial interviews and 24 students who responded to the follow-up survey. (See Table 1: Participant Demographics)

**Table 1:** Participant Demographics

	Averages for medical students years 1–3 N27	First year medical students N10	Second year medical students N10	Third year medical students N7
Average Age	26	25	27	28
Gender	78% F 19% M 3% non-binary	90% F 10% M	71% F 14% M 14% Non-binary	70% F 30% M
Ethnicity	White: (22%) Hispanic (37%) Asian (22%) Black: (4%) Native American: (4%) Two or more ethnicities: (11%)	40% White 30% Hispanic 10% Hispanic/Native American 10%, Asian 10%, Filipino	14% Asian 14% Black 14% White Hispanic 29% Hispanic 14% Native American/Hispanic 14% Chinese American	20% White 40% Hispanic 10% Latina 10% Chinese 10% Native American 10% Asian
Relationship status	Single: 33% In a relationship 56% Married: 11%	Single: 40% In a relationship 60% Married: None	Single: 29% In a relationship 57% Married: 14%	Single:30% In a relationship 50% Married: 20%
Children	11% have children	None	2 students	1
Parental Responsibilities	Less than 1% is a caretaker for ca. parent	No	No	1
Living situation	Lives alone: 22% Lives with roommate: 15% Lives with partner renting: 33% Lives with partner owns home: 4% Lives with parent or other family members: 26%	Lives alone: 20% Lives with roommate: 30% Lives with partner renting: 20% Lives with partner owns home: 10% Lives with parent or other family members: 20%	Lives alone: 29% Lives with partner renting: 29% Lives with parent or other family members: 42%	Lives alone: 20% Lives with roommate: 10% Lives with partner renting: 50% Lives with parent or other family members: 20%

### General happiness and satisfaction

On average, participants rated themselves as a slightly happier person and identified slightly more with the characterization of “enjoying life regardless of what is going on” rather than “someone who never seems as happy as they might be.” They also considered themselves slightly happier when compared to most of their peers. (See Table 2: General Happiness of Participants.

**Table 2:** Initial self-rated happiness of participants

Averages for medical students years 1–3 N28	First year medical students N10	Second year medical students N8	Third year medical students N10
**Q1: In general, I consider myself**: **(scale of 1–7, with 1 = not a very happy person, 7 = a very happy person)**			
Average: 5.4 Ranged from 2–7	Average: 5.7 Ranged from 5–7	Average: 5 Ranged from 3–6	Average: 5.5 Ranged from 2–7
**Q2: Compared to most of my peers, I consider myself**: **(scale of 1–7, with 1 = less happy, 7 = more happy)**			
Average: 4.9 Ranged from 2–7	Average: 5.2 Ranged from 4–6	Average: 4.5 Ranged from 3–7	Average: 5.0 Ranged from 2–7
**Q3: Some people are generally very happy. They enjoy life regardless of what is going on, getting the most out of everything. To what extent does this characterization describe you at this point in time?** **(scale of 1–7, with 1 = not at all, 7 = a great deal)**			
Average: 4.9 Ranged from 2–7	Average: 4.8 Ranged from 2–7	Average: 4.6 Ranged from 3–7	Average: 5.3 Ranged from 2–7
**Q4: Some people are generally not very happy. Although they are not depressed, they never seem as happy as they might be. To what extent does this characterization describe you at this point in time? (scale of 1–7, with 1 = not at all, 7 = a great deal)**			
Average: 2.6 Ranged from 1–7	Average: 3.1 Ranged from 1–5	Average: 3.6 Ranged from 2–6	Average: 2.1 Ranged from 1–7

### Learning satisfaction

Participants were slightly dissatisfied with the number of hours spent on zoom since the start of their online curriculum. Third year participants were slightly dissatisfied with the school’s approach to helping their wellness during the pandemic compared to the first- and second-year cohorts who reported on average as somewhat satisfied. Overall, participants were slightly satisfied with the synchronous learning and somewhat satisfied with the asynchronous learning. See Table 3: Learner satisfaction with online curriculum.

**Table 3:** Learner satisfaction with online curriculum

	Overall averages for medical students years 1–3 N28	First year medical student average N10	Second year medical student average N8	Third year medical student average N10
How do you feel about the number of hours you spend on zoom since the start of your online curriculum?	3.7	3.5	4.3	3.6
Are you satisfied with the school’s approach to helping your wellness during the pandemic?	4.6	5.0	5.3	3.6
How satisfied are you with synchronous learning?	4.6	4.5	4.7	4.6
How satisfied are you with asynchronous learning?	5.1	4.8	5.2	4.5

Medical students in this study perceived that they have three identities, that of an individual person, as a learner, and as a future medical professional. Covid-19 affected all three. On an individual level, first year medical students had to pivot quickly to adjust to a new environment - medical school – along with expectations that were not met. For example, expectations for how learning would occur. However, those that did express feelings of isolation within the school setting also expressed either the positives that resulted from isolation such as appreciating social interactions when they did occur or resiliency. *“… nothing went like I had planned in the beginning and anticipated. Anatomy was something I had looked forward to doing - and the virtual lab they had us doing was NOT what I was looking for when I started medical school. I came into medical school not knowing anybody, and I thought we would be going to lectures, and doing study groups and I would meet people that way. So, being entirely on Zoom at home was disappointing. But, our on-line group has slowly expanded. There are still people I haven’t met in my class. But now I’m feeling better - I feel we have developed a small group that works really well. And I think that’s what I expected - it’s hard but it’s manageable. (MS1)”*.

Medical students in their second year expressed positive effects of online learning such as enjoying being at home and the ability to be more productive and reconnect with family. Two parents in the second year cohort expressed difficulty at the beginning of the pandemic due to lost childcare and less time for themselves.

Third year students noted feelings of restriction and sadness from not being able to see do things they used to enjoy or to see family and friends regularly. One student noted that “*it has forced me into isolation, not being able to hang out with friends like before*. (MS3)”

### Education disrupted…

Academically, the themes for all three cohorts were individual preference for learning styles. Zoom worked well for those who identified as enjoying solitary learning and are self-motivated. Other students had to re-adjust their learning styles, cope with unmet expectations of what medical school would be like and often felt disconnected from others, from learning, and from the school as a whole. One second year student felt the impact of COVID in the academic realm more than personally or professionally, *“I probably [saw] the most impact on this one. I used to go in-person every day and it was hard when the pandemic hit. During CVPR [Cardiovascular, Pulmonary, and Renal block], it was really hard to transition online but in the end it was fine. It was very stressful and it was an emotional roller coaster. I would get really nauseous at one point just looking at my computer and shifting to my books.(MS2)”*

It was remarked by at least two students in each cohort that medical school requires in-person training for certain skills and exams, “ *I think medical school and lots of the expectations for beginning this kind of journey fell by the wayside. Everything I thought it was going to feel and be like, it was different. Also pretty hard to engage in some more serious and emotional parts….It’s very hard to share and communicate important issues like cultural competency…. I feel that certain subjects don’t have the same effects over this digital platform. I don’t think it’s an intimate form of communication when you’re trying to talk about emotional or intimate subjects. (MS1)”*

### Creating a professional identity

While several positives such as the ability to learn more about public health and epidemiology than anticipated, the predominant theme amongst first year medical students was anxiety about the inability to develop a professional identity. Three of the students remarked on the imposter phenomenon resulting from isolation and lack of in-person events and training. One student remarked that, *‘I think it’s (the pandemic) made me less professional - I feel less like a student-doctor. I feel like I let my guard down in how I talk to my peers. I forget that they’re going to be my professional peers some day. I forgot not to be myself.(MS1)”* While the imposter phenomenon was not directly mentioned by second years, it was clear that there was a lack of opportunities to work directly with patients and clinicians and “*learning to collaborate professionally has been lost. (MS2)”* This is a deficit in professional education even while the opportunities to explore other areas such as research have been gained, “*I did not get as much clinical experience as I wanted to this past year. I got involved in more research experience instead.(MS2)”* Third year students felt a professional impact with shortened clinical experiences which made deciding on a future specialty difficult. There was a sense of uncertainty and “. *still (not knowing) what kind of doctor I want to be*. (MS3)” Another student remarked “*I feel like it would be much harder for me to choose what I want to do since I will only be getting a snapshot of the specialties. A couple weeks rotating through one specialty does not provide me with enough information on whether I would feel comfortable pursuing that as a career. (MS3)”*

### Achieving and maintaining wellness in a disrupted academic environment

One consistent theme as the pandemic continued, was a feeling of weariness and not being able to achieve wellness. Concrete active sessions such as cooking, yoga, art and counseling were universally remarked upon and linked back to satisfaction with the school’s approach. Wellness resources that are more social in nature tend to be best remembered. Because free counseling services and wellness checks provided by mental health professionals were the wellness activities participants were most aware of and used at least once since the pandemic started, particularly by the first year cohort, there is an opportunity to re-consider wellness resources offered by medical students. For example, having access to the school’s gym (the gym was closed at the beginning of the pandemic due to social distancing rules) or community clubs that offered recreational group activities like hiking. Several first-year students suggested that receiving regular check-ins by faculty or the office of wellness on campus could benefit student wellness. Within all three cohorts, participants expressed the importance of physical health and having optional, rather than mandatory, wellness sessions provided by the school.

With all three cohorts, there were mixed feelings with regards to satisfaction with the school’s approach to wellness. While many were satisfied with the school’s emphasis on wellness and knew how to access wellness resources and were appreciative of efforts, there was a sense of things being done to simply “check a box” without “meaningful change.” As one student stated, *“I was very impressed during orientation about how much wellness seemed to matter - balance and taking good care of yourself- but I have not seen any follow through whatsoever. (MS1)”* Similarly, another student commented that “*I felt like there were too many emails promoting the idea/theory of wellness. I did not see any actions actually taken by the school to provide wellness or to increase wellness in their students*. (MS3)”

### What is stressing medical students out? Another triparate state of individual circumstances, academics, and the world at large

Causes of stress varied amongst the three cohorts. However, predominant themes revolved around the medical school curriculum, home environment, and political climate. First-year students felt stressed by the overwhelming load and expectations of medical school. One student commented *“my expectations of myself are changing a lot. It’s different in med school - you try so hard and you never get what you want academically. It hurts a lot, I get down a lot. (MS1)*” Throughout the three cohorts, participants experienced stress with changes in their home environment as “*there’s no delineation because school and home*. (MS1)” Participants worried about the health and safety of their family members with the ongoing Covid pandemic. A father in the second-year cohort felt stress about the balance between school and child-care, noting *“it flipped my life upside down and doubled my workload. (MS2)”* Regarding the political climate, several students felt distressed when thinking about the election and protests.

Slightly more than three-fourths (78%) of participants reported better or no change in physical health since March 2020. Reasons for improved changes in health included healthier eating, eating regularly, improved sleep, and seeking care. Two participants experienced unintentional weight gain or fluctuations, leading to reported decreased health. A first year student noted flare ups of her interstitial cystitis with increased stress. A third year student reported more back problems.

Changes in mental health were varied amongst the three cohorts. Four first year students experienced more anxiety and worry related to school and family members’ health during the pandemic. In the second year cohort, responses were mixed with two students reporting depression or anxiety and two students reporting improved mental health. In the third year cohort, five students expressed stable or no changes in their mental health while three students reported improved mental health.

Participants managed their stress in a variety of ways. Most participants engaged in exercise and physical activity. Other strategies included spending time with family and friends, reaching out to faculty for support, and medicating for psychiatric disorders. Specific coping mechanisms utilized included:

1. Exercise: Many participants in the first and second year cohort reported a decrease in the amount of daily exercise with predominant reasoning including gym closures and less free time due to school work, while 50% of third-year students reported an increase in exercise and 30% reported no change in amount of exercise.
2. Diet: Amongst the three cohorts, changes in diet varied. Several students reported healthier eating habits like eating a vegetarian diet and more home-cooked meals. Many students reported their diet worsening due to eating more convenient pre-packaged foods, using take-out services, snacking, or overeating.
3. Sleep: Ten participants reported no change in sleep, 9 reported increased sleep, and 8 reported decreased, irregular, or worsened sleep. No second-year student reported increased sleep, but 57% reported decreased or worsened sleep. In contrast, no third-year student reported decreased or worsened sleep with 60% of students reporting no change in sleep.
4. Other: Praying, having a positive mindset, increased communication, therapy, journaling, seeking help, reading, and meditation. Unhealthy behaviors adopted included changes in sleep habits (e.g. sleeping in or sleeping late), increased screen time, and poor dietary habits (e.g. eating more prepared foods, sugars/sweets, comfort foods, snacking). Negative coping mechanisms employed included indulging in food and decreased social interactions.

### The importance of relationships during disruptions

For most participants, relationships with others improved. Relationships and communication improved for students who lived with their parents, family members, or significant others due to having more time at home together. However, four students did note that their relationship with their significant other or parents did suffer initially due to the unfamiliar stress and tension from being at home all the time together. For students that did not live with their parents or other family members, relationships grew distant. Two students remarked that relationships with friends suffered primarily due to lack of communication and inability to be as involved with one another.

Was COVID related stress in medical student really the primary driver of unhappiness? While averages do not account for individual circumstances, it is clear that life events beyond universal experience of COVID does affect self-perceived happiness. In general, 1st and 2nd year medical students became happier over the year, while 3rd year students became slightly more unhappy. See Table 4: One year follow-up of self-rated happiness.

**Table 4:** One year follow-up of self-rated happiness

First year medical students		Second year medical students		Third year medical students	
Pre-Covid Average N10	Post-Covid Average N10	Pre-Covid Average N8	Post-Covid Average N6	Pre-Covid Average N10	Post-Covid Average N9
**Q1: In general, I consider myself**: **(scale of 1–7, with 1 = not a very happy person, 7 = a very happy person)**					
Average: 5.7	Average: 5.6	Average: 5	Average: 5.2	Average: 5.5	Average: 5.4
**Q 2: Compared to most of my peers, I consider myself**: **(scale of 1–7, with 1 = less happy, 7 = more happy)**					
Average: 5.2	Average: 5.2	Average: 4.5	Average: 4.4	Average: 5.0	Average: 5.1
**Q3: Some people are generally very happy. They enjoy life regardless of what is going on, getting the most out of everything. To what extent does this characterization describe you at this point in time?** **(scale of 1–7, with 1 = not at all, 7 = a great deal)**					
Average: 4.8	Average: 4.8	Average: 4.6	Average: 4.8	Average: 5.3	Average: 4.8
**Q4: Some people are generally not very happy. Although they are not depressed, they never seem as happy as they might be. To what extent does this characterization describe you at this point in time?** **(scale of 1–7, with 1 = not at all, 7 = a great deal)**					
Average: 3.1	Average: 2.7	Average: 3.6	Average: 3.4	Average: 2.1	Average: 2.3

## Discussion

There were personal losses that affected all respondents. Divorces, change in living conditions, loss of employment in order to start school, missed graduations, missed trips, and missed in-person educational experiences. These losses may be felt more acutely when they are positive expectations that have been denied. According to some cognitive theories [36], happiness is a reflection of “discrepancies between perceptions of life as it is and notions of how life should be. First year students had expectations for what their education “should look like”, yet COVID-19 was a disrupter. True to this theory, there is also evidence for the assumption that standards adjust over time and that effects of life-events on happiness are therefore short lived which may account for the slight variations in self-reported happiness students as they adjust to this new “reality”.

Academically, there were pivoting expectations within the educational setting from anticipated in-person learning with peer interaction to almost exclusive online learning. This adjustment requires an ability to quickly adapt one’s learning style as well as physical space in which to learn which can cause considerable stress, particularly among first year students who are new to medical school and third years who were supposed to begin clinical rotations. This situational stress is consistent with what has been noted in health professional students around the world [37]. However, there were also positive experiences. Positivity is notable in mitigating stress and offers an opportunity for a shift in focus from what cannot be controlled to what can be influenced. On a global level, online education removed many barriers to collaboration and allowed students an opportunity to collaborate with others more frequently which contrasts to our findings (Table 3: learmer satisfaction with online curriculum) where online learning was really only enjoyed by some [38].

The results from this study suggest that the COVID-19 pandemic and its associated changes in the learning format and environment may create a new risk factor in the development of imposter syndrome as trainees perceive that some critical skills in medicine are best learned in the context of in-person learning which was not available to them. Happiness as a sense of self and who you “should be” was challenged during the pandemic. There is a growing body of research on the imposter syndrome that suggests that it is common in physicians and physicians in training, ranging from 22–60% and is associated with increased rates of burnout and suicide [39]. Research outside the context of the pandemic has suggested that comprehensive changes in medical education and culture will be necessary to change this phenomenon [40, 41]. Longitudinal research on physicians in training during the COVID-19 pandemic will be critical to understand the long-term impact on imposter syndrome and may require intervention above and beyond current efforts to address imposter syndrome.

A notable strength of this study is the large representation of groups underrepresented in medicine, with 78% of the participants self-identifying as Hispanic, Native American, Asian, Black, or multi-racial. This representation is especially important given recent findings that medical students who are underrepresented in medicine experience higher levels of exhaustion-related burnout [42]. In addition, while some quantitative studies have assessed happiness as a construct in medical students during covid (e.g., [43], this mixed method study explored the unique individual experiences medical students faced during the COVID-19 pandemic and how those experiences inform their opinions of their institution and their own reports of well-being, happiness, and mental health.

### Limitations

Although it is not the primary goal of our study, one of the main limitations is there is no definitive answer as to whether happiness or lack thereof, is directly correlated to the pandemic which was felt by all or whether happiness is caused by external events more than personal ability to retain a positive outlook in the midst of stressful events. Another limitation is the participants were interviewed by peers, albeit of a different cohort. This may cause underreporting of negative events and negative coping strategies and personal well-being outcomes. This may also cause some students to overreport positive feelings and coping strategies. However, we believe our strategy of mixing interviewer/interviewee schooling levels and recruiting volunteers rather than a convenience sample alleviates some of these possibilities. Finally, this is a single site study with 10% representing an entire cadre of students.

## Conclusion

While changes in learning due to the COVID-19 pandemic negatively impacted medical students’ lives across domains, it also provided opportunities traditionally not available during training. More time at home, more flexibility with time, opportunities to connect with household members, and increased access to mental health resources were all seen as positives for some participants. The results from this study suggest that the COVID-19 pandemic and changes in the learning format and environment may create a new risk factor in the development of imposter syndrome. Opportunities to leverage these positive aspects should be considered in medical student education post-pandemic.

## Data Availability

The datasets generated and/or analysed during the current study are not publicly available due to individual privacy concerns, but are available from the corresponding author on reasonable request.
